# Tylvalosin Tartrate Improves the Health Status of Swine Herds during Immunization with Porcine Reproductive and Respiratory Syndrome Virus-Inactivated Vaccine

**DOI:** 10.3390/vetsci10010012

**Published:** 2022-12-25

**Authors:** Qianru Zhang, Chenchen Cui, Siyu Zhang, Xiaohong Deng, Xuehui Cai, Gang Wang

**Affiliations:** 1 State Key Laboratory of Veterinary Biotechnology, Harbin Veterinary Research Institute, Chinese Academy of Agricultural Sciences, Harbin 150069, China; 2 College of Veterinary Medicine, Xichang University, Xichang 615000, China; 3 Shandong Provincial Key Laboratory of Animal Biotechnology and Disease Control and Prevention, College of Veterinary Medicine, Shandong Agricultural University, Taian 271018, China

**Keywords:** tylvalosin tartrate, PRRSV-inactivated vaccine, immune responses

## Abstract

**Simple Summary:**

Variants of porcine reproductive and respiratory syndrome virus (PRRSV) strains complicate measures taken to control PRRS. In recent years, tylvalosin tartrate has been used to control PRRSV infection in swine farms as well as to improve the swine herd’s status during PRRSV vaccination. To investigate the effect of tylvalosin tartrate during PRRSV-inactivated vaccine immunization, we fed piglets a diet medicated with tylvalosin tartrate during PRRSV-inactivated vaccine immunization. The cumulative data showed that tylvalosin tartrate attenuated the increase in total white blood cells induced by immunization at day one post-immunization (DPI), induced an increase in monocyte counts after seven DPI, and attenuated the intensity of the inflammatory response induced by vaccination and increased IFN-γ expression at three and seven DPI. In addition, the administration of tylvalosin tartrate could also attenuate the reduction in the percentage of CD8^+^ T cells induced by PRRSV-inactivated vaccine immunization at seven DPI. These results demonstrated that the combination of inactivated PRRSV vaccines with tylvalosin tartrate could improve the function of the immune system in piglets and improve the health status of swine herds during PRRSV-inactivated vaccine immunization.

**Abstract:**

Porcine reproductive and respiratory syndrome (PRRS) is a devastating disease that affects pigs and is responsible for severe economic losses. The commercial PRRSV-inactivated vaccine (CH-1a strain) in China was recently selected to control PRRS in large populations of PRRS-positive sows and was found to effectively reduce the rate of stillbirth abortion based on clinical observations. However, stress from vaccine inoculation (e.g., fever, anorexia, abortions, and slow body weight gain) usually appears after immunization on many swine farms. In this study, we fed piglets a diet medicated with tylvalosin tartrate during PRRSV-inactivated vaccine immunization. We found that tylvalosin tartrate attenuated the increase in total white blood cells induced by immunization at day one post-immunization (DPI) and induced an increase in monocyte counts after seven DPI. There was also attenuation in the intensity of the inflammatory response induced by vaccination and elevation of serum IFN-γ concentrations at three and seven DPI after immunization. The administration of tylvalosin tartrate could also attenuate the reduction in the percentage of CD8^+^ T cells induced by PRRSV-inactivated vaccine immunization at seven DPI. These results demonstrated that in addition to tylvalosin tartrate being able to control respiratory and enteric bacterial infections in swine farms, it can also improve the stress status of swine herds during PRRSV-inactivated vaccine immunization.

## 1. Introduction

Porcine reproductive and respiratory syndrome (PRRS) is caused by the PRRS virus (PRRSV) [1,2]. It represents a devastating disease that affects pigs and is responsible for severe economic losses to swine farms worldwide [3,4,5,6]. The typical clinical symptoms of PRRS include mild to severe respiratory disease in infected newborns and growing pigs, as well as reproductive failure in pregnant sows, depending on the virulence of the PRRSV strain, as well as the immune status of infected pigs [1,7,8,9,10]. Although the first PRRSV strain (Ch-1a) was isolated in 1996, PRRS began to impact pig herds before this time in China [11]. After the strain was isolated, Ch-1a-inactivated and CH-1R-modified live vaccines (MLV) were developed based on the dominant PRRSV strains in 2005 and 2007 [12], respectively.

Ch-1R MLV has made substantial contributions to the prevention and control of PRRS in swine herds since 2006, when a highly pathogenic PRRS (HP-PRRS) epidemic disease broke out in China [8,13,14]. Since the end of 2019, strains of the African swine fever virus (ASFV) of different virulence appeared on swine farms [15,16], accompanied by infection with NADC30/34-like strains in swine herds [17,18,19]. PRRSV MLVs, including HP and classical PRRSV MLV vaccination, can cause disease through feedback from personnel staff, which includes fever, anorexia, and respiratory symptoms in piglets, as well as a reproduction disorder in sows. Since then, the PRRSV Ch-1a-inactivated vaccine has been recognized after a dormancy period of approximately 15 years.

In China, the efficacy of the PRRSV-inactivated vaccine is controversial since no experimental data are available to support the protection of this vaccine against the PRRSV challenge in PRRSV-free piglets [20,21,22,23,24]. However, based on clinical observations, the inactivated PRRSV CH-1a vaccine can effectively control the abortion of PRRSV-positive unstable sow herds, and was advertised by some manufacturers of the PRRSV CH-1a inactivated vaccine and in lots of PRRSV-positive unstable sow herds. Due to the status differences in various swine farms, signs of vaccine inoculations stress (e.g., fever, anorexia, abortions, and slow body weight gain) typically appear after immunization.

The active ingredient of tylvalosin tartrate is the tartrate salt of tylvalosin, which belongs to macrolide, and was authorized for pigs in the EU in 2004 [25]. Tylvalosin is currently used to control respiratory and enteric bacterial infections in poultry and swine herds [26]. In addition, the administration of tylvalosin tartrate can enhance macrophage phagocytic activity, as well as reduce oxidative stress and decrease pro-inflammatory cytokine expression [27]. From the clinical observation, tylvalosin tartrate has also recently been shown to control PRRSV infection in swine farms, as well as improve the swine herd’s status during PRRSV vaccination (both MLVs and inactivated vaccines) by dietary tylvalosin tartrate supplementation in the feed. 

In this study, we investigated the effect of tylvalosin tartrate during PRRSV-inactivated vaccine immunization. The results showed that tylvalosin tartrate could attenuate the increased whole white blood cell count induced by immunization and induce an increase in monocyte counts. Tylvalosin tartrate has also been found to attenuate the intensity of the inflammatory response induced by vaccination and elevate the serum IFN-γ concentrations after immunization. Moreover, the administration of tylvalosin tartrate can also attenuate the reduction in the percentage of CD8^+^ T cells induced by PRRSV-inactivated vaccine immunization. These results demonstrate that tylvalosin tartrate can attenuate stress status and improve the health status of swine herds during PRRSV-inactivated vaccine immunization.

## 2. Materials and Methods

### 2.1. Ethical Statement 

The animal experiment protocols were approved by the Animal Ethics Committee of Shandong Agricultural University (Approval Number: #SDAUA-2021-P009). The methods were conducted in accordance with the approved animal ethics guidelines.

### 2.2. Animal Experimental Design

A total of 20 21-day-old PRRSV-free commercial breed piglets (the body weight ranged from 4–7 kg for each piglet) were randomly divided into four groups (5 piglets/group) of group A (tylvalosin tartrate + PRRSV-inactivated vaccine group), group B (PRRSV-inactivated vaccine group), group C (tylvalosin tartrate) and group D (negative control group). The weaned piglets at 21 days old were numbered by ear-tagging and acclimated for 1 week before experiments. In groups A and C, the piglets were fed a diet containing 800 mg/kg Aivlosin (20% Tylvalosin Tartrate Premix, Zhejiang ECO-BIOK Animal Health Products Co., Ltd., Zhejiang), whereas the others were fed an unmediated diet. After 7 days, the piglets from groups A and B received a single dose of the PRRSV-inactivated vaccine (CH-1a strain) intramuscularly (i.m.). The treated pigs received the medicated feed until 14 days post-vaccination. The piglets in group D received no medication or vaccine as the negative control. Throughout the duration of the study, all animals received food and water ad libitum. The clinical symptoms and rectal temperatures of all experimental piglets were recorded each day after immunization.

### 2.3. Blood Sample Collection

Blood samples were collected at −7, 0, 1, 4, 7, 14, and 21 DPI to evaluate the effect of the tylvalosin tartrate on the pigs following administration of the PRRSV-inactivated vaccine. Briefly, 5 mL of blood was drawn from the precaval vein of all piglets, 3 mL of blood was put into blood collection tubes (Jiangsu, Yuli medical instrument) which contained gel a with coagulant to separate the serum from the whole blood, and another 2 mL of blood was put into blood collection tubes (AOSAITE, Shandong) containing EDTAK2 to prevent clotting. The serum samples were stored in 1 mL aliquots at −80 °C until further use.

### 2.4. Serum Cytokine Detection

Commercially available ELISA kits (Cusabio, Wuhan, China) were used to determine the concentrations of IL-1β and IFN-γ in serum in accordance with the manufacturer’s instructions. The intra- and inter-assay coefficients of variation for assays are CV% < 8% and CV% < 10%, respectively.

### 2.5. Detection of the Proportion of Peripheral Blood Leukocytes and Lymphocytes

An amount of 50 μL of whole blood containing EDTAK2 was used to analyze the T lymphocytes cell subset changes in PBLs. CD3^+^ cells, CD4^+^CD8^−^ cells, and CD4^−^CD8^+^ cells were labeled with anti-porcine CD3ε-SPRD MAb (PPT3, Southern Biotech, AL, USA), anti-porcine CD4-FITC MAb (74-12-4, Southern Biotech AL, USA), and anti-porcine CD8-R-PE MAb (76-2-11, Southern Biotech AL, USA), as described previously [13], and analyzed by flow cytometry (FCM, BD, NJ USA). Another 2 mL of whole blood was used to analyze the types of leukocytes in the whole blood using an Automatic Blood Cell Analyzer (IDEXX, Westbrook, Maine 04092 USA).

### 2.6. Statistical Analysis

The numerical data are presented as the mean ± S.D. and were analyzed using GraphPad Prism software (version 5.02 for Windows; GraphPad Software Inc.). Differences between the groups were assessed using Wilcoxon rank sum tests or a one-way ANOVA and Tukey multiple-comparison test. A *p*-value less than 0.05 was considered to be statistically significant.

## 3. Results

### 3.1. Clinical Presentation

**Figure 1:**
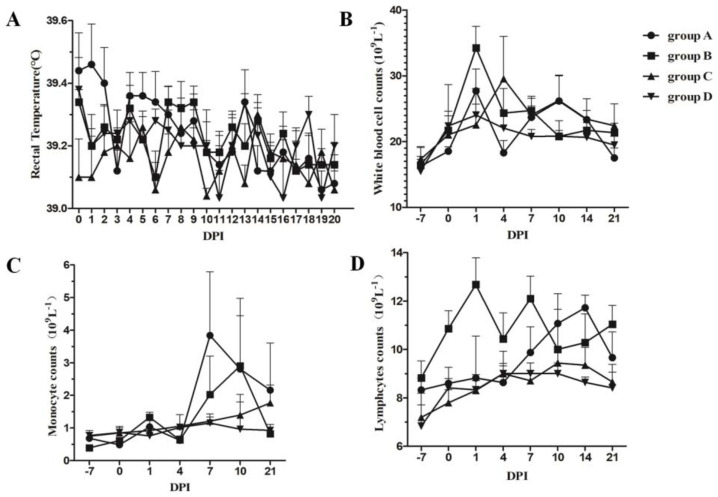
**Changes in rectal temperature (A) and leukocyte counts in the peripheral blood (B–D) during PRRSV-inactivated vaccine immunization.** Group A and group B piglets were immunized with 1 dose/pig of the PRRSV Ch-1a-inactivated vaccine on 0 DPI, and group A and group C piglets were fed a diet containing 800 mg/kg Aivlosin (20% Tylvalosin Tartrate Premix) from −7 to 14 DPI, respectively. Analysis of the white blood cell count (**B**), monocyte counts (**C**), and lymphocyte counts (**D**) in the peripheral blood. Each number represents the mean ± standard deviation (S.D.) generated from all piglets on each DPIs.

To judge the different clinical presentations among the four groups, the clinical course was monitored during the experiment. Rectal temperature was recorded every day in the morning from 0 to 20 DPI (Figure 1A), and no piglets exhibited fever in any of the four groups. Two piglets in group D (control group) displayed severe lethargy, anorexia, and emaciation due to bacterial infection and were humanely euthanized at seven and ten DPI, respectively. The other piglets in the four groups showed no clinical signs during the experiment.

### 3.2. Dynamic Changes in White Blood Cell Counts

White blood cells can defend against pathogen infection or other foreign antigens by processing infectious agents and producing antibodies. Immunization with the Ch-1a-inactivated vaccine also induced changes in white blood cell counts, which included the total white blood cells, monocytes, and lymphocyte counts. The changes in white blood cells are shown in Figure 1B–D, where it can be seen that immunization with the inactivated vaccine induced increased total white blood cells at one DPI; however, tylvalosin tartrate attenuated this immunization-induced increase. No difference was observed regarding changes in white blood cells at other time points. Immunization with the inactivated vaccine also induced an increase in monocyte counts from seven DPI, and tylvalosin tartrate elevated the increase in these cells. Furthermore, immunization with the inactivated vaccine induced an elevation in lymphocytes from 1 to 21 DPI and peaked at 12.8 × 10^9^ cells/L. The administration of tylvalosin tartrate delayed the increase to 7 DPI and persisted to 21 DPI.

### 3.3. Dynamic Changes in Serum IL-1β and IFN-γ

**Figure 2:**
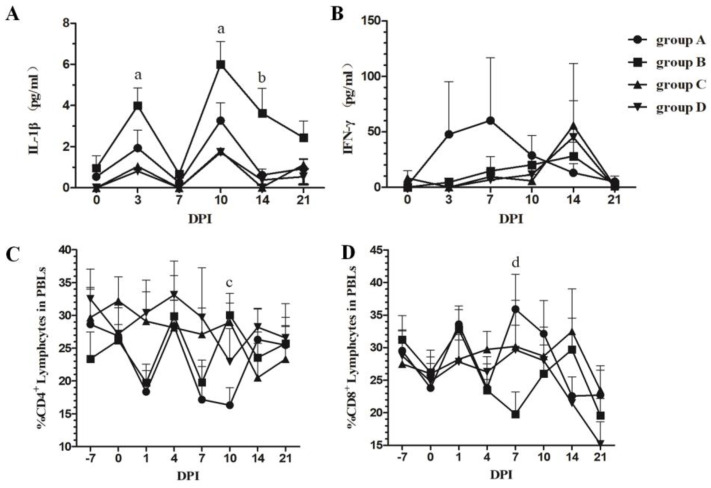
Concentrations of serum IL-1β and IFN-γ and changes in the percentage of T lymphocyte subsets in PBLs. Serum concentrations of IL-1β (**A**) and IFN-γ (**B**) were measured using commercial ELISA kits (Cusabio, China); Changes in the T lymphocyte subset in PBLs for CD4 (**C**) and CD8 (**D**) expression were detected by flow cytometry. Each point represents the mean value (±S.D) generated from each group on different DPIs. a, group B is higher significantly than groups C and D (*p* < 0.05); b, group B is higher significantly than groups A and C (*p* < 0.05); c, group A is lower significantly than groups B and C (*p* < 0.05); d, group A is higher significantly than group B (*p* < 0.05).

The concentrations of serum IL-1β and IFN-γ represent the inflammatory reaction and immune response induced by inactivated PRRSV vaccine immunization, respectively. Dynamic changes in serum IL-1β and IFN-γ were investigated using the commercially available ELISA kits. Figure 2A,B shows that immunization up-regulated the concentrations of IL-1β from 3 to 21 DPI (except 7 DPI); however, tylvalosin tartrate attenuated the intensity of the inflammatory response induced by vaccination during the experiment. While vaccine immunization alone could not induce IFN-γ expression, tylvalosin tartrate increased the concentrations of IFN-γ at three and seven DPI after immunization. 

### 3.4. Changes in the Number of CD4^+^CD8^−^ and CD4^−^CD8^+^ T Cells in PBLs

Alterations in the CD4^+^ and CD8^+^ T cell populations of peripheral blood lymphocytes (PBLs) were detected to evaluate the cellular immune response during the experiment. As shown in Figure 2C,D, immunization with the CH-1a-inactivated vaccine reduced the percentage of CD4^+^ T cells at one DPI and seven DPI and was not affected by tylvalosin tartrate administration. Moreover, immunization also reduced the percentage of CD8^+^ T cells at seven DPI, but the changes could be attenuated by combining vaccination with tylvalosin tartrate administration.

## 4. Discussion

In China, PRRS has been widespread for more than 20 years. Since the first PRRSV isolate Ch-1a was reported in 1996, the PRRS epidemic has never been stopped. In 2006, the emergence of a highly pathogenic PRRSV strain threatened the swine industry in China [8,13,14]. More recently, the PRRSV strains NADC30 and NADC34 were recently introduced into China and have undergone mutations or recombination with local PRRSV strains in swine farms in China [18,28,29,30,31]. This has resulted in variant viruses that have led to various outcomes, ranging from inapparent to severe. Despite a range of effects and diverse approaches, several challenges remain for the control of PRRS [32,33,34,35]. Following the emergence and prevalence of naturally occurring, less-virulent African swine fever viruses (ASFVs) in domestic pigs in China since 2020 [16], many pig herds continue to be at risk when vaccinated with a PRRSV MLV due to a modulated immune response by the less-virulent ASFV [16,36]. As a result, even though there were no experimental animal data to prove the protective effect of PRRSV-inactivated vaccine against any epidemic PRRSV isolate challenges, the only commercial PRRSV-inactivated vaccine (CH-1a strain) in China was still selected to control PRRS in large numbers of PRRS-positive sows and effectively reduce the rate of stillbirth abortion collected from clinical observations. However, multiple negative side effects, including anorexia, fever, and abortion, were often observed during PRRSV-inactivated vaccine immunization, and tylvalosin tartrate has been chosen for use on a widespread basis to improve the health status of swine herds.

Tylvalosin tartrate has been recognized to possess a broad spectrum of biological activities usually used for therapeutic applications to respiratory and enteric bacterial infections (*Lawsonia intracellularis, Mycoplasma hyopneumoniae*, and *Pasteurella multocida*, etc.) in pigs to reduce the level of pathogen infection and the occurrence lung lesions and to improve the average daily feed consumption and feed conversion efficiency, etc. [26,37]. Tylvalosin tartrate has also been reported to exhibit anti-inflammatory properties in different models, including PRRSV infection, which decreased IL-8, IL-6, IL-1β, and TNF-alpha concentrations induced by lipopolysaccharide (LPS) or PRRSV treatment in vitro or in vivo [27,38]. In this study, PRRSV-free piglets were selected for inoculation with the PRRSV-inactivated vaccine, and the obvious side effects (e.g., fever and anorexia) induced by vaccination were not observed. However, as a foreign antigen, PRRSV-inactivated vaccine administration also induced IL-1β responses from 3 to 21 DPI (except 7 DPI) (Figure 2A, group B), and tylvalosin tartrate was able to modulate the intensity of the inflammatory response induced by vaccination (Figure 2A, group A). These results are consistent with previous reports of the anti-inflammatory property of tylvalosin tartrate in different models [38].

White blood cells can defend against pathogen infection or other foreign antigens by processing infectious agents and producing antibodies. Different types of stimulation (including vaccination stress) are characterized by either an increased or decreased number of granulocytes, lymphocytes, or monocytes, which represents part of the complex defense mechanism against different antigens. Immunization with the Ch-1a-inactivated vaccine also induced changes in white blood cell counts (the total white blood cells, monocytes, and lymphocyte counts). Therefore, we investigated the different changes in immune cell counts among each of the four groups. During the experiment, the white blood cell counts within the peripheral blood were the greatest in the group that received the inactivated vaccine alone, but when tylvalosin tartrate was incorporated into the treatment, the whole white blood cell counts decreased. This finding indicated that inoculation with inactivated vaccines can induce a robust white blood cell response that can be improved by tylvalosin tartrate. Previous studies have reported that macrolide antibiotics can be used as immunomodulatory medications to promote monocyte-to-macrophage differentiation, increase the killing ability of macrophages, and stimulate phagocytic chemotaxis [39]. Monocytes are an important type of antigen-presenting cell (APC) that play a key role in building a bridge for innate and adaptive immune responses [40]. In this experiment, tylvalosin tartrate upregulated the population of the monocytes and lymphocytes from 7 DPI, which maybe contribute to eliciting the immunological response to the PRRSV-inactivated vaccine. The regret is that it is difficult to induce obvious antibody responses after only one round of inactivated vaccine immunization, and we did not evaluate the effect that tylvalosin tartrate has on vaccine titers following vaccination.

Correspondingly, by detecting the percentage of CD4^+^ and CD8^+^ T cells among peripheral blood lymphocytes, we found that tylvalosin tartrate did not affect changes in CD4^+^ T cells; however, it could attenuate the decrease of CD8^+^ T cells during immunization with the inactivated CH-1a vaccine. Tylvalosin tartrate also elevates serum IFN-γ concentrations during immunization (Figure 2B), which may help to activate macrophages, cytotoxic T cells, and NK cells [41]. The cumulative results demonstrated that in addition to the original effect of tylvalosin tartrate in controlling *Mycoplasma hyopneumoniae* infection in swine farms, it can also improve the pig body’s immune responses to the foreign antigens (e.g., the PRRSV Ch-1a-inactivated vaccine).

During the experiment, two of the piglets in the control group were humanely euthanized at 7 and 10 DPI due to bacterial infection. We collected the related data from three other piglets in the control group, which satisfied the need to make a comparison with the other groups. In addition, there were no experimental data to support the efficacy of the PRRSV-inactivated vaccine against PRRSV infection in PRRSV-free piglets, so we only investigated how the tylvalosin tartrate attenuated vaccine inoculation stress (e.g., fever, anorexia, abortions, and slow body weight gain) observed in swine herds after immunization. The mechanism of the PRRSV-inactivated vaccine is still unclear, so we did not carry out further research to assess the differences that occur by combining the inoculation of the inactivated PRRSV vaccine and tylvalosin tartrate administration and then performing a PRRSV challenge on the piglets.

## 5. Conclusions

In conclusion, tylvalosin tartrate was widely chosen to control vaccine inoculation stress during PRRSV-inactivated vaccination. It can attenuate the inflammation induced by vaccination as well as improve the body’s immune status by regulating immune cell counts. Thus, tylvalosin tartrate fed to piglets during PRRSV-inactivated vaccination can effectively attenuate inoculation stress as well as control infections from common bacteria such as *Lawsonia intracellularis* and *Mycoplasma hyopneumoniae* in swine herds, thus improving the health status of these herds.

## Data Availability

Not applicable.
